# Methacrylated poly(glycerol sebacate) as a photocurable, biocompatible, and biodegradable polymer with tunable degradation and drug release kinetics

**DOI:** 10.21203/rs.3.rs-3384762/v1

**Published:** 2023-10-06

**Authors:** Mei-Li L. Bice, Valentina L. Ortega, Marina H. Yu, Kevin J. McHugh

**Affiliations:** Rice University

## Abstract

Poly(glycerol sebacate) (PGS) is a biodegradable, elastomeric polymer that has been explored for applications ranging from tissue engineering to drug delivery and wound repair. Despite its promise, its biomedical utility is limited by its rapid, and largely fixed, degradation rate. Additionally, its preparation requires high temperatures for long periods of time, rendering it incompatible with heat-sensitive molecules, complex device geometries, and high-throughput production. In this study, we synthesized methacrylated PGS (PGS-M), imparting the ability to rapidly photocross-link the polymer. Increasing the degree of methacrylation was found to slow PGS-M degradation; PGS-M (5.5 kDa) disks with 21% methacrylation lost 43% of their mass over 11 weeks in vivo whereas 47% methacrylated disks lost just 14% of their mass over the same period. Increasing the methacrylation also extended the release of encapsulated daunorubicin by up to two orders of magnitude in vitro, releasing drug over months instead of one week. Like PGS, PGS-M exhibited good biocompatibility, eliciting limited inflammation and fibrous encapsulation when implanted subcutaneously. These studies are the first to perform long-term studies demonstrating the ability to tune PGS-M degradation rate, use PGS-M to release drug, demonstrate sustained release of drug from PGS-M, and evaluate PGS-M behavior in vivo. Taken together, these studies show that PGS-M offers several key advantages over PGS for drug delivery and tissue engineering, including rapid curing, facile loading of drugs without exposure to heat, tunable degradation rates, and tunable release kinetics, all while retaining the favorable biocompatibility of PGS.

## Introduction

**Figure 1:**
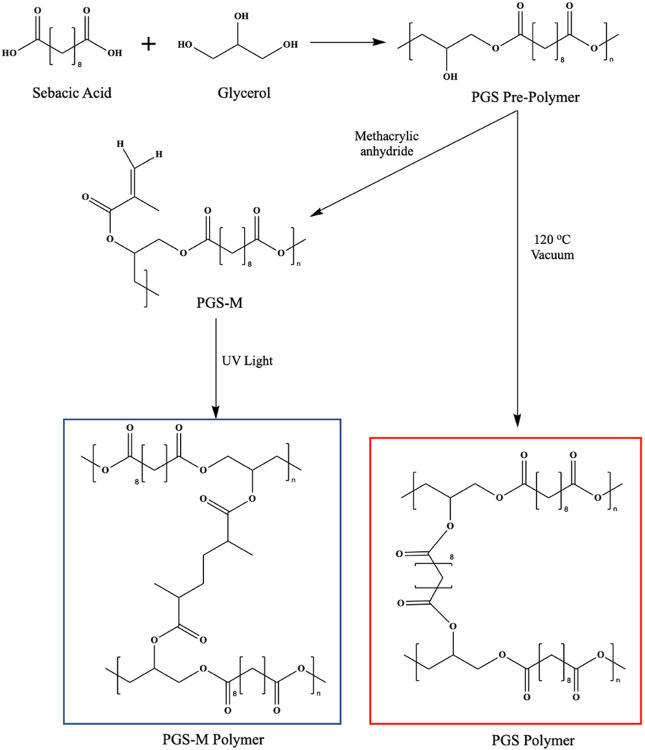
Reaction schemes for PGS pre-polymer synthesis and PGS modification with methacrylic anhydride, and the molecular structures of photocured PGS-M (blue box) and heat- and vacuum-cured PGS (red box).

Polyglycerol sebacate (PGS) is an elastomeric polymer composed of sebacic acid, a naturally occurring fatty acid metabolite, and glycerol, a building block of lipids [1, 2]. PGS is attractive as a biomaterial due to its biocompatibility, biodegradability, and tunable mechanical properties, rendering it useful across a wide range of biomedical applications, including tissue engineering, surgical wound repair, and drug delivery. Most widely explored for tissue engineering applications, PGS has often been used as a scaffold to support the growth of soft tissues, including myocardium [3-5], adipose tissue [6], cartilage [7-10], and blood vessels [11]; it has also been investigated as a patch that delivers cardiac cells to infarcted regions of the heart [12], as a conduit for peripheral nerve repair [13], and as a scaffold for retinal progenitor cells in the subretinal space [14]. Though reported on less than tissue engineering, PGS has also been used to extend the release of cancer therapeutics and antibiotics [15-17]. PGS is synthesized in a facile two-step process involving the polycondensation of glycerol and sebacic acid at 120°C for 24 hr followed by cross-linking at 120°C under strong vacuum (Fig. 1). Altering certain reaction parameters during PGS synthesis, namely the reaction time, temperature, and ratio of glycerol to sebacic acid, provide the ability to control pre-polymer molecular weight, mechanical properties, and behavior [9, 12, 18, 19].

Despite the favorable features of PGS, its biomedical applications are restricted by its limited compatibility with biological molecules and tissue during synthesis and device formation. Cross-linking PGS pre-polymer requires high temperature (> 110°C), strong vacuum (< 5 Pa), and long reaction times (> 24 hr) [20, 21], which prevents its potential for direct polymerization in tissue, restricts the geometries it can assume, and damages heat sensitive molecules (e.g., proteins and some small molecules) encapsulated within the polymer network. These limitations make PGS sub-optimal for tissue engineering and drug delivery applications, which often benefit from specific device structure and/or the integration of drugs, such as growth factors and antibodies. Several groups have sought to overcome these limitations by modifying PGS to form a photocurable polymer without exposure to high temperatures, low pressures, or long reaction times. By functionalizing the free secondary hydroxyl group on PGS with either an acrylate or methacrylate, PGS-acrylate (PGS-A) or PGS-methacrylate (PGS-M) can be produced [22, 23]. When exposed to ultraviolet (UV) light in the presence of a photoiniator, these polymer systems can be photocured in seconds to minutes, eliminating the need for harsh curing conditions. Importantly, this method of curing allows for the incorporation of heat-sensitive molecules into the polymer network and permits the creation of complex geometries with light-based methods, such as 3-D printing and photolithography as well as rapid in-mold curing. PGS-M is particularly appealing since, unlike PGS-A synthesis, it does not require the use of acryloyl chloride, which would otherwise generate a substantial quantity of chlorine salts which can be toxic; additionally, the presence of highly reactive acrylates renders the polymer susceptible to spontaneous cross-linking prior to use, affecting processability [22, 23].

Previous attempts at controlling the degradation rate of PGS, namely by altering the molecular weight and curing time, have been largely unsuccessful [23, 24]. As a result, most studies have seen PGS degrade completely within 60 days [13, 20, 24-27]. Although the lifetime of PGS devices could likely be prolonged by increasing their thickness, this approach is highly problematic for many tissue engineering applications that seek to recapitulate native tissue dimensions as well as for controlled drug delivery applications in which injectability through a standard needle is desirable. The purpose of this study was to identify the parameters that impact in vitro and in vivo PGS-M behavior, namely the influence of the degree of methacrylation and molecular weight on the degradation rate and drug release profiles. Importantly, we assessed the in vivo biocompatibility of PGS-M, which, to our knowledge, has not been previously reported on in literature. Finally, we evaluate the in vitro release of a model drug from PGS-M matrices to better understand the correlation between degradation, drug loading, and release kinetics for drug delivery applications.

## Materials/Methods

### PGS Pre-Polymer Synthesis

PGS pre-polymer was synthesized by combining equimolar amounts of glycerol and sebacic acid under nitrogen at 120°C for 24 hr, at which point water was removed from the reaction. It was then placed under vacuum (20 mbar) for a further 32, 46, and 58 hr to yield pre-polymers with different molecular weights (MW). Pre-polymer MWs were assessed via advanced permeation chromatography (APC) at 30 mg/mL in tetrahydrofuran (Waters Corp., Milford, MA, Acquity APC System, XT 125, 2.5 μm, 4.6 x 150 mm column).

### PGS-M Synthesis

PGS pre-polymer was weighed in a round bottom flask, dissolved in dichloromethane at a 1:4 ratio (w/v) and cooled to 0°C in an ice bath. To calculate the amount of methacrylic anhydride needed for each reaction, it was assumed that the primary hydroxyl groups in glycerol reacted with sebacic acid, leaving one secondary hydroxyl, or 3.9 mmol of hydroxyl groups per gram of PGS pre-polymer, available for functionalization. Different quantities of methacrylic anhydride (0.21–0.70 mol hydroxyl group/mol of polymer) were added dropwise to the round bottom flask followed by an equimolar amount of triethylamine and 4-methoxyphonel (0.01 wt%) to prevent spontaneous crosslinking. The reaction was allowed to return to room temperature with constant stirring for 24 hr. For purification, the solution was washed twice with hydrochloric acid (30 mM) at a 1:1 ratio. The aqueous portion of the mixture was then removed, and the remaining organic layer was dried with granular calcium chloride (CaCl_2_). The entire solution was vacuum-filtered and the dichloromethane was removed with a rotary evaporator. The resulting pre-polymer was stored in sealed vials, protected from light, at 4°C for up to 1 week prior to use.

### Methacrylation Analysis

PGS pre-polymer and PGS-M pre-polymer were assessed by ^1^H nuclear magnetic resonance spectroscopy (NMR) (Bruker, Billerica, MA, 600 MHz NMR Spectrometer). Samples were prepared in deuterated chloroform (1% w/v) and analyzed with TopSpin software. The CDCl_3_ reference peak was identified at 7.25 ppm. The peaks at 1.25(8H, s, methylene), 2.39 (4H, br, methylene), and 1.62 ppm (4H, br, methylene) correspond to sebacic acid protons and the peaks at 4.20 (4H, br, methylene) and 5.37 (1H, s, methine) ppm correspond to glycerol protons. The peaks at 1.90 (3H, d, methyl), 6.19 (2H, d, methylene), and 5.61 (H, d, methylene) are associated with the protons of the PGS-M pre-polymer methacrylate group; the degree of methacrylation was calculated by comparing the signal integral at 1.90 ppm to the sebacic acid peak at 1.25 ppm.

### PGS-M Disk Fabrication

To create photocured disks, PGS-M pre-polymer was combined with diphenyl (2,4,6-trimethylbenzoyl) phosphine oxide/2-hydroxy-2-methylpropiophenone (1% w/w) and methanol (50 μL per gram of polymer) and mixed vigorously for 2 min. The solution was then placed under house vacuum to remove methanol. Using a spatula, a small amount of PGS-M pre-polymer was carefully placed on a clean glass microscope slide and pressed between another slide separated by 380 μm polytetrafluoroethylene (Teflon) shims on either side. The entire apparatus was placed under house vacuum to remove any air bubbles and residual methanol and then exposed to UV light using an Omnicure S2000 (Excelitas Technologies Corp., Waltham, MA) with a collimating adapter (350–700 nm) (Thorlabs Inc., Newton, NJ) at 10% intensity for 1 min to photocure the PGS-M pre-polymer. The glass slides were carefully separated leaving a 380 μm-thick sheet of cured PGS-M on one of the glass slides. A circular biopsy punch with a 6.35 mm diameter was then used to cut thin disks from the cured PGS-M sheet.

### Uncured Material

PGS-M disks approximately 6.35 x 0.38 mm were weighed (initial mass), placed in 2 mL of methanol overnight in a closed container, removed from methanol, dried under house vacuum at room temperature for 3 hr, and then weighed again (remaining mass). The uncured content was calculated using the following formula:

$$UncuredContent\%=100x\frac{InitialMass-RemainingMass}{InitialMass}$$

Cured polymer content, therefore, was calculated as:

CuredContent%=100−uncuredcontent%

### In Vitro Degradation of PGS-M Disks

Photocured PSG-M disks free of uncured polymer were weighed to obtain their initial mass after methanol soaking and then placed in either 1 mL of phosphate-buffered saline (PBS) or 1 mL of PBS with 40 units/mL cholesterol esterase (CE) from porcine pancreas (Alpha Diagnostics, San Antonio, TX); each solution contained 1% (v/v) penicillin-streptomycin. The disks were agitated for 48 hr on an orbital shaker at 80 RPM, removed from solution, rinsed with PBS, dried at 70°C for 3 hr, weighed to assess cumulative mass loss (formula below), and placed back into fresh media.


$$CumulativeMassLoss\;(\%)=100x\frac{InitialMass-RemainingMass}{InitialMass}$$

### In Vitro Release of Daunorubicin and Drug Loading

Freshly prepared photocured PGS-M disks were placed overnight at room temperature in methanol containing 20 or 60 mg/mL of daunorubicin. The disks were carefully removed from solution, dried under house vacuum for 3 hr to remove methanol, and then rinsed in PBS to remove adsorbed daunorubicin. To determine the amount of daunorubicin initially loaded into the disks, drug-loaded disks of each PGS-M polymer (n = 3) were dissolved in 2,2,2-trifluoroethanol (5 mL) for 24 hr. The resulting solution was then diluted and analyzed for fluorescence (480/590 nm excitation/emission) using a Tecan Infinite 200Pro microplate reader (Tecan Group Ltd., Männedorf, Switzerland). Concentration was calculated using a daunorubicin standard curve and then converted to loading based on each disk’s mass.

To assess daunorubicin release, drug-loaded disks were placed in either 1 mL of PBS or 1 mL of PBS containing 40 units/mL of CE; each solution also contained 1% (v/v) penicillin-streptomycin. After 48 hr of incubation at 37°C, the supernatant was collected and daunorubicin concentration was determined via the fluorescence analysis technique described above.

### PGS Hydrophobicity Analysis

The relative hydrophobicity/hydrophilicity of each PGS-M polymer, both non-drug loaded and drug-loaded, was assessed using an OCA 15EC optical contact angle goniometer (Data Physics Instruments, Charlotte, NC). A sessile drop of deionized water (5 μL, dispensing rate of 3 μL/s) was placed on a flat surface of PGS-M polymer. Using dpiMAX software, the contact angle was measured continuously for 200 seconds and the angle at 180 seconds was reported.

### In Vivo Degradation

#### Disk Preparation

Within a sterile environment, PGS-M disks were soaked in methanol for 24 hr, dried, and weighed to determine their initial mass. The dimensions of each disk were also assessed with digital calipers before being placed in a sterile 24-well microplate. In total, 8 disks for each group and timepoint were prepared for analysis: five for degradation/microscopy analysis and three for histological evaluation.

#### Surgical Implantation

All surgical procedures were approved by the Institutional Care and Use Committee of Rice University prior to study onset and performed according to the NIH Guidelines for the Care and Use of Laboratory Animals. Sprague-Dawley male and female rats were first weighed and then anesthetized with 1–4% continuous inhalation of isoflurane. The back of the animal was then shaved with an electric razor and scrubbed with betadine followed by ethanol (70% v/v). Next, an injection of meloxicam and bupivacaine was given near the anticipated incision site for prophylactic pain relief. While under isoflurane, a small midline incision was made down the animal’s back. Using blunt dissection tools, four subcutaneous pockets were created in the upper and lower left and right spaces relative to the incision. A single PGS-M disk was placed in each pocket and the incision was closed with several skin clips. To achieve experimental independence, a maximum of one of each type of PGS-M disk was implanted into an individual animal.

#### Surgical Explantation

Groups of animals were euthanized after 1, 3, 5, 7, and 11 weeks via carbon dioxide inhalation and the disks were removed from the surrounding tissue capsule for weight loss/microscopy. Disks were rinsed in PBS, dried at 70°C for 6 hr, and then weighed. To remove adsorbed biological materials, the disks were soaked in SDS (2% w/v) overnight, rinsed in Milli-Q water overnight, and then dried at 70°C for 6 hr.

Disks for histological evaluation were carefully excised, keeping the tissue capsule and surrounding structures intact, and then fixed in 10% buffered formalin at room temperature overnight. Next, samples were transferred to a 30% sucrose solution and stored at 4°C overnight before being placed in cryomolds filled with optimal cutting temperature (OCT) compound. Samples were sliced, stained with either hematoxylin and eosin (H&E) or Masson’s Trichrome, and imaged at the MD Anderson Research Histology Core Laboratory.

#### Statistics

Multiple group comparisons were calculated with one-way or two-way ANOVA with Tukey’s multiple comparison test. Student’s T test was used to test differences between two groups. The linearity of daunorubicin release from PGS-M was assessed using linear regression analysis. Calculations were performed in GraphPad Prism 9. The threshold for statistical significance was set at p < 0.05. The degree of statistical significance is denoted with asterisks as follows: * = *p* ≤ 0.05; ** = *p* ≤ 0.01; *** = *p* ≤ 0.001; **** = *p* ≤ 0.0001.

## Results/Discussion

### Materials Characterization of PGS-M

PGS-M was synthesized by combining equimolar amounts of sebacic acid and glycerol at 120°C under vacuum for up to 58 hr. Portions of the polymer product were removed from the reaction vessel after 32, 46, and 58 hr, which resulted in weight average molecular weights (MW) of 3.2, 5.5, and 8.9 kDa, respectively. While other papers have explored the use of PGS with much higher MWs (e.g., 15 kDa and above), we chose to use lower MW PGS (< 10 kDa) for our studies as they typically exhibit less branching, making them more consistent to synthesize and amenable to functionalization; additionally, lower MW is less viscous prior to photocross-linking and therefore easier to process.

**Figure 2:**
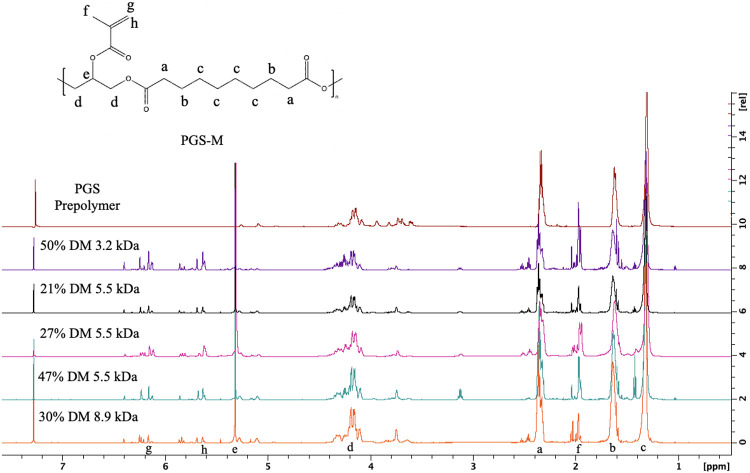
Proton NMR analysis of PGS and PGS-M pre-polymers with varying MWs and DMs. The sebacic acid protons in PGS and PGS-M correspond to a (2.39 ppm), b (1.62 ppm), c (1.25 ppm), and glycerol peaks are found at d (4.20 ppm), and e (5.37 ppm). PGS-M’s methacrylate protons are labeled f (1.90 ppm), g (6.19 ppm), and h (5.61 ppm).

PGS pre-polymers were methacrylated to produce PGS-M pre-polymers with varying degrees of methacrylation (DM). As demonstrated by NMR analysis (Fig. 2), the PGS pre-polymer contains distinct peaks at 1.25, 1.62, 2.39, 4.20, and 5.37 ppm. However, methacrylating PGS with methacrylic anhydride produces 3 distinct peaks at 1.90, 6.19, and 5.61 ppm that are not observed in the original PGS pre-polymer spectra. To calculate the DM, the integral of the sebacic acid peak at 1.25 ppm was compared to the integral of the 1.90 ppm methacrylate peak.

We synthesized a library of pre-polymers with various MWs and DMs, namely, 3.2 kDa with 30% and 50% DM, 5.5 kDa with 21%, 27%, 47% and 60% DM, and 8.9 kDa with 30% and 50% DM by varying the reaction conditions. Of those materials, we found that the 5.5 kDa pre-polymer with 60% DM and the 8.9 kDa with 50% DM began curing during the final rotary evaporation step of synthesis without the addition of a photoinitiator or exposure to high-intensity light, rendering them unusable. We suspect that exposure of these highly methacrylated systems to ambient light induced free-radical polymerization promoted by the presence of reactive methacrylate groups available for cross-linking [28], an anoxic environment (oxygen is known to inhibit free radical polymerization) [29], and the application of heat during evaporation, which increases the rate of polymerization [30].

**Figure 3:**
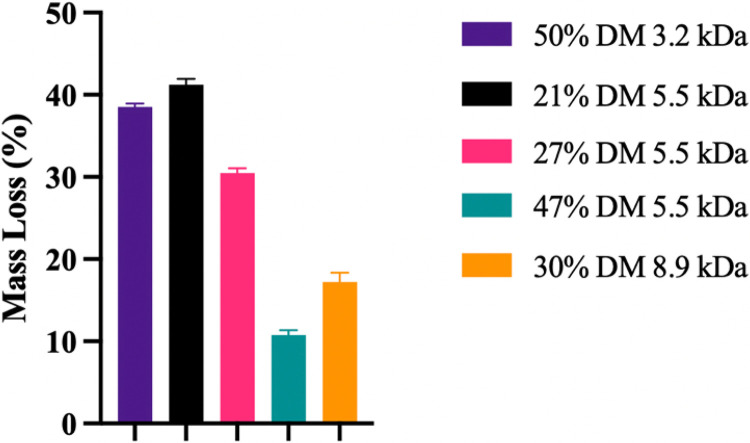
Graph showing the effects of DM and MW on the percentage of PGS-M that remains unintegrated and methanol-soluble after photocross-linking. Data is represented as the mean ± standard deviation (n=6). The differences in mass loss were statistically significant for all groups (p<0.0001).

In addition to chemical characterization by proton NMR, the physical properties of the PGS-M polymers were assessed by examining the uncross-linked material after UV curing. As seen in Fig. 3, all polymers tested were found to have statistically different amounts of uncross-linked material (p < 0.0001). For polymers of the same MW, increasing the DM significantly reduced the amount of the polymer that did not integrate into the solid after photocuring. This is expected since increasing the number of methacrylate groups per polymer chain increases the number of potential cross-links that polymers can form to integrate into the cured network, preventing removal during the subsequent methanol soak [31]. For polymers with different MWs but similar DMs, for example, 50% DM 3.2 kDa vs 47% DM 5.5 kDa, the lower MW polymer had more uncured content at 38.5 ± 0.4% vs 10.8 ± 0.6% (p < 0.0001), which can likely be attributed to the lower number of methacrylate groups on the shorter polymer chains despite having the same DM. This trend is also observed when comparing the 27% DM 5.5 kDa vs 30% DM 8.9 kDa PGS-M with uncured mass loss of 30.5 ± 0.6% vs 17.2 ± 1.1% (p < 0.0001). Taken together, the data suggests that both the DM and MW strongly impact cross-linking density and the residual amount of uncured polymer after UV exposure.

Lacking sufficient methacrylation also caused issues as the 30% DM 3.2 kDa PGS-M did not cure under UV light exposure, suggesting it did not meet the minimum cross-linking density to form a solid polymer network. Analyzing the number of methacrylate groups per polymer chain using a combination of NMR data and MW, the 30% DM 3.2 kDa polymer was calculated to contain approximately 3.98 methacrylate groups per polymer chain. In comparison, the material with the lowest methacrylates per polymer chain that we tested and confirmed to be capable of photocuring was the 21% DM 5.5 kDa PGS-M, which contained approximately 4.78 methacrylates per polymer chain, suggesting that there is likely a minimum threshold necessary for materials to cure into a solid polymer network. The other curable forms of PGS-M, 50% DM 3.2 kDa, 21%, 27%, and 47% DM 5.5 kDa, and 30% DM 8.9 kDa, contained 6.64, 4.78, 6.14, 10.69, and 11.10 methacrylate groups per polymer chain, respectively. This underscores the importance of attaining a sufficiently high combination of MW and DM to generate PGS-M with substantial mechanical properties.

### In Vitro Degradation

The in vitro degradation of PGS-M was assessed to determine the impact of MW and DM on polymer degradation rate. Polymers were placed in either phosphate-buffered saline (PBS) or PBS containing 40 units/mL cholesterol esterase (CE) to emulate in vivo enzymatic degradation, as done by other studies [22, 23, 32]. Pancreatic CE has been shown to degrade polyesters in vitro [33, 34] and is similar to esterases secreted by macrophages that contribute to polyester degradation in vivo [35]. Of the five polymers and two release conditions evaluated, only 21% DM 5.5 kDa PGS-M showed meaningfully accelerated release in the earlier segment of the release assay, losing 24.8 ± 2.8% of its initial mass over the first six days before losing an additional 27.2 ± 6.7% over the next 32 days. For the other materials and conditions, the rate of degradation was largely consistent over the study duration. 21% DM 5.5 kDa PGS-M was also the fastest degrading polymer, losing 1.37% of its mass per day in CE when averaged over the duration of the experiment. At the opposite end of the spectrum, three polymers (50% DM 3.2 kDa, 47% DM 5.5 kDa, and 30% DM 8.9 kDa) lost mass at a rate of < 0.1% per day. Taken together, these results suggest that degradation could potentially be tuned from months to years.

For 5.5 kDa PGS-M disks, increasing the DM decreased the rate of degradation. Compared to the 27% and 47% DM 5.5 kDa polymer, the 21% DM 5.5 kDa polymer disks degraded rapidly and were structurally fragile at all time points, weakening to the point of fracture on day 38. On day 38, the 21%, 27%, and 47% DM 5.5 kDa polymers lost 52.0 ± 8.7%, 9.2 ± 0.9%, and 3.7 ± 1.2% of their masses, respectively. This corresponds to 27% DM 5.5 kDa PGS-M degrading nearly six-fold slower than the 21% DM polymer and the 47% DM polymer degrading approximately 14-fold slower than the 21% DM polymer.

In contrast, MW did not seem to play a large role in determining degradation rate–at least within the conditions tested. 3.2 kDa and 5.5 kDa PGS-M disks with similar DM (50% and 47%) lost 5.3 ± 0.6% and 3.7 ± 1.5% of their masses, respectively, after 40 days in CE, which was not statistically significantly different. 8.9 kDa and 5.5 kDa PGS-M disks with similar DM (30% and 27%) degraded 4.4 ± 0.9% and 8.7 ± 1.35%, respectively, after 40 days in CE, which is statistically significant (p < 0.05); however, this difference may be largely due to the slight difference in DM, which is important in this range, as demonstrated by the aforementioned six-fold increase in degradation rate observed for the 5.5 kDa PGS-M when the DM decreases just 6% from 27% DM to 21% DM.

The inclusion of CE in the release media did not uniformly accelerate PGS-M degradation. Many factors have been shown to influence the enzymatic degradation of methacrylated polymers including the DM, cross-linking density, and device morphology [36, 37]. The 50% DM 3.2 kDa polymer degraded much more slowly than the 21% DM 5.5 kDa polymer despite their similarities in the amount of uncured material and relatively similar numbers of methacrylate groups per polymer chain, which might have otherwise suggested that they would degrade at similar rates [23, 38]. Therefore, these metrics do not appear to be predictive of the degradation rate. We hypothesize that the high DM for the 50% DM 3.2 kDa polymer induced a tightly bound, highly cross-linked structure that sterically prevented the comparatively large (67 kDa) CE enzyme from readily accessing and cleaving ester bonds, thereby slowing degradation. The methacrylate cross-linker used in our studies is relatively short, which would exacerbate the effects of this proposed mechanism [39, 40]. Furthermore, the degradation rates of PGS-M polymers, except for the 21% DM 5.5 kDa polymer, were nearly identical in PBS with and without CE suggesting that CE had minimal impact on the degradation rate of the polymers, consistent with the hypothesis that increasing the DM decreases enzyme access to the polymer’s ester bonds.

### In Vitro Drug Release

Based on the results of the degradation assays, the 21, 27, and 47% DM 5.5 kDa PGS-M polymers were chosen for in vitro drug release studies. PGS-M’s ability to swell in organic solvents after photocross-linking was used to load drug into the swollen polymer [17], which avoided exposing the encapsulated drug to UV light. heat, or reactive groups. Each polymer disk was soaked overnight in methanol containing 20 mg/mL daunorubicin, removed from solution, washed, dried, and assessed for drug loading. These studies showed that 21, 27, and 47% DM 5.5 kDa disks were loaded with 10.5, 7.5, and 1.7% (w/w) daunorubicin, respectively. To increase loading of the 47% DM 5.5 kDa polymer, a second set of disks was soaked in a higher concentration daunorubicin solution (60 mg/mL), yielding drug loading of 5.7% (w/w). Incubating these disks in PBS with or without CE resulted in largely linear drug release from every PGS-M polymer evaluated over the duration tested. The 27% DM 5.5 kDa polymer incubated in CE fractured rapidly, breaking into small pieces by day 32. Based on the degradation study data, the 21% DM 5.5 kDa polymer would likely have also fractured, though release was completed before that occurred. All other disks maintained their structural integrity throughout the 60-day study.

**Figure 6:**
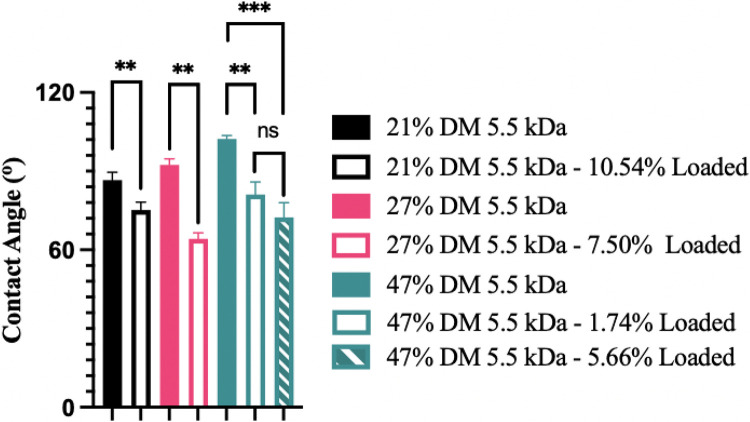
Hydrophobicity of 5.5 kDa PGS-M disks with different DM and daunorubicin loading levels (w/w). Average contact angle ± standard deviation reported (n=5). **p<0.01, ***p<0.001, ns = not significant.

Mirroring the in vitro degradation data, the 21% DM 5.5 kDa PGS-M disks had a faster rate of drug release than the 27% and 47% DM 5.5 kDa polymers. For all three polymers, drug released faster than degradation progressed, which has been observed by other groups for many other materials, including unmodified PGS [16]. This is not unexpected as drug release typically exceeds mass loss due to the degradation of the surrounding polymer matrix. Other important factors such as drug diffusion and leaching as well as polymer morphology have been reported to strongly impact the rate of drug release [41, 42]. The addition of daunorubicin, a water-soluble, hydrophilic drug, to the matrix may have increased the rate of water infiltration into the polymer, potentially facilitating drug release [43] and promoting the fracture of 27% DM 5.5 kDa PGS-M disks. This hypothesis is supported by contact angle analysis (Fig. 6), which shows that the addition of daunorubicin increased the hydrophilicity of the PGS-M disks.

Similar to other studies with different polymers, we found that increasing the concentration of drug in the matrix increased the rate of drug release [16, 44]. By day 60, the 47% DM 5.5 kDa disks loaded with more daunorubicin (5.7%) released approximately 30% of their drug payload compared to only 10% of the drug payload being released by the same disks loaded with less daunorubicin (1.7%). This could, in part, be a consequence of increased device hydrophilicity with drug loading as the contact angle was observed to decrease with increased drug loading in the 47% DM 5.5 kDa PGS-M group. Together, these data suggest that drug loading significantly impacts a drug’s release profile from PGS-M and could be another parameter capable of modifying release and/or degradation rate.

### In Vivo Degradation

**Figure 7:**
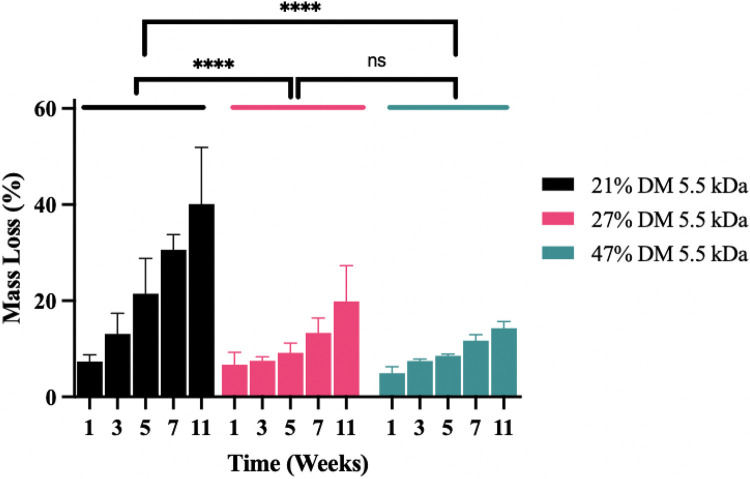
In vivo mass loss from 5.5 kDa PGS-M disks with different DM after 1, 3, 5, 7, and 11 weeks compared to in vitro mass loss at 1, 3, 5, and 7 weeks. In vivo data reported as averages ± standard deviation (n=5). In vitro degradation data in CE reported as averages ± standard (n=3). ****p<0.0001, ns=not significant.

To assess in vivo performance, 21, 27, and 47% DM 5.5 kDa PGS-M disks were surgically implanted into the subcutaneous space on the flanks of Sprague-Dawley rats, explanted at 1, 3, 5, 7, and 11 weeks, and weighed to assess in vivo polymer degradation. The data show that the 21% DM 5.5 kDa PGS-M disks degraded at a significantly faster rate than both the 27% and 47% DM samples (p < 0.0001), as expected, but that the 27% and 47% DM samples had rates of in vivo degradation that were not statistically significantly different from one another (Fig. 7). The rate of disk mass loss was relatively constant for the samples after an initial period of accelerated degradation immediately following implantation.

Methacrylating PGS appeared to be an effective method of slowing its degradation rate in vivo. As discussed previously, unmodified PGS’s utility for soft tissue engineering applications and implants has been stifled by its rapid in vivo degradation. In one of the earliest studies using PGS for biomedical applications, Wang, et al. observed complete in vivo degradation of PGS disks (12.5 mm diameter, 2 mm height) within 60 days [27]. Similarly, Pomerantseva, et al. saw complete in vivo resorption of PGS disks (10 mm diameter, 5 mm height) by 5 weeks [24]. In contrast, our studies found that photocross-linked PGS-M exhibited much slower in vivo degradation with 57.1 ± 11.5%, 82.7 ± 5.7%, and 85.7 ± 1.1% mass remaining 11 weeks after implantation for the 21 %, 27%, and 47% DM 5.5 kDa disks, respectively. We suspect that this change is due to substantial differences between traditionally cured PGS and PGS-M structures. The PGS-M cross-linker is a shorter chain length compared to PGS’s cross-linker which is comprised of sebacic acid and glycerol (Fig. 1). The shorter cross-linker in PGS-M likely produces a more densely compacted network of polymer chains, limiting access of esterases to cleavable ester bonds and depending more on hydrolysis for degradation. In contrast, thermally cured unmethacrylated PGS contains long cross linkers that can expose cleavable esters to esterases.

Evaluating these polymers in vivo was especially critical since differences between in vitro and in vivo results are very common [24, 45-48]. In vitro, the 27% DM 5.5 kDa disks lost mass approximately twice as fast as 47% DM 5.5 kDa PGS-M disks, whereas these polymers lost mass at nearly the same rate in vivo. Degradation also progressed more quickly in vivo than it did in PBS containing CE in vitro. In contrast, the 21% DM 5.5 kDa sample degraded more rapidly in vitro than in vivo (p < 0.05). This discrepancy might be explained by the inability to completely mimic the relative in vivo contributions of hydrolysis and esterase activity in vitro. Interestingly, disks implanted in vivo had no integrity issues at any time point studied. Disks comprised of 21% DM 5.5 kDa PGS-M, which all fractured within 5 weeks in vitro, maintained their structural integrity in vivo through at least 11 weeks. As a result, a comparison of the in vivo and in vitro studies demonstrates that, although using CE in the release media more closely mimics the in vivo microenvironment than PBS alone, it is not a perfect model.

### Biocompatibility Evaluation

The same subcutaneous implantation model was used to assess the tissue response to PGS-M in vivo. The tissue surrounding the implanted disks were removed at 1, 3, and 7 weeks, sectioned, and visualized by H&E and Masson’s Trichrome staining. The appearance of the surrounding tissue remained largely unchanged over the 7-week time course. Namely, inflammation was minimal at all times investigated, as indicated by a lack of macrophage accumulation at the tissue-disk interface. Fibrous capsule formation was also minimal, which suggests that PGS-M, like unmodified PGS [19, 20], exhibits favorable biocompatibility.

## Conclusions

The purpose of this study was to identify the factors affecting the in vitro and in vivo degradation rates of PGS-M, evaluate PGS-M biocompatibility, and examine the drug release profiles that can be obtained. The results from this study indicate that PGS-M releases drug in a relatively linear fashion and that the rate of drug release can be altered by changing the polymer’s DM and drug loading. Overall, PGS-M’s rate of degradation is substantially slower than reported from conventionally prepared PGS in vitro and in vivo. These features highlight the potential of PGS-M to be used for long-term drug delivery and tissue engineering applications on the order of months to years, overcoming a limitation of traditional PGS. Furthermore, PGS-M is photocurable, providing the potential to create complex scaffold structures through 3D photolithographic techniques and directly incorporate thermosensitive drug molecules into the polymer scaffold with post-curing loading. Finally, PGS-M was shown to cause only a minimal inflammatory response, another key factor in determining clinical relevance. Taken together, this paper establishes the in vivo compatibility of PGS-M and highlights its potential utility for both tissue engineering and drug delivery applications.

## Figures and Tables

**Figure 4:**
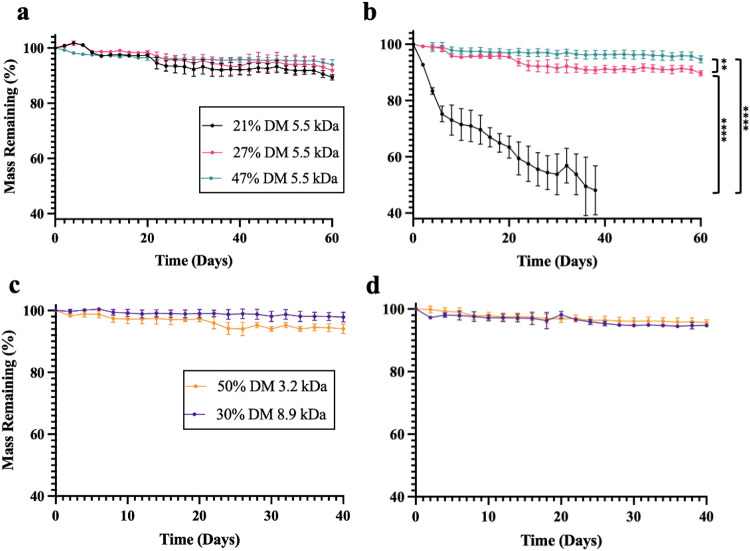
In vitro degradation of PGS-M polymers. Data represented as the mean percentage of mass remaining ± standard deviation (n=3). A and B correspond to 21, 27, and 47% DM 5.5 kDa PGS-M degradation at 37 °C in PBS and PBS with CE, respectively. C and D correspond to 50% DM 3.2 kDa and 30% DM 8.9 kDa mass loss at 37 °C in PBS and PBS with CE, respectively. **p<0.01, ****p<0.0001.

**Figure 5:**
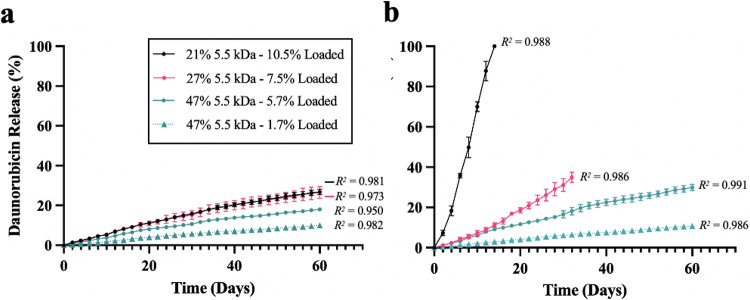
Cumulative daunorubicin release represented as percentage release over time in (A) PBS or (B) PBS containing CE. Points show actual data, lines depict linear regressions. Error bars indicate average ± standard deviation (n=3). R^2^ values indicate how well the data fit to a linear regression.

**Figure 8:**
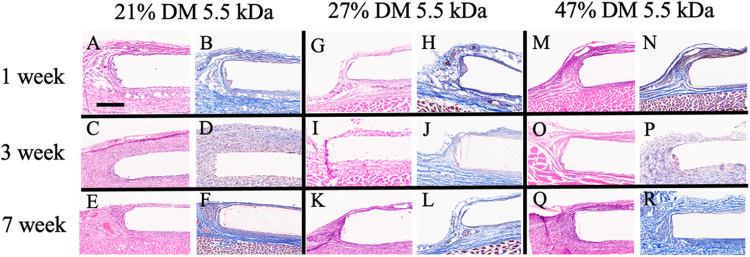
In vivo histological evaluation of the PGS-M implant site over a seven-week timeframe. Representative images of H&E and Masson’s Trichrome staining for 21% DM 5.5 kDa (A-F), 27 % DM 5.5 kDa (G-L), and 47% DM 5.5 kDa (M-R) (n=4). Scale bar = 350 mm.

## Data Availability

The datasets generated during and/or analyzed during the current study are available from the corresponding author on reasonable request.
